# CCR8 is expressed by post-positive selection CD4-lineage thymocytes but is dispensable for central tolerance induction

**DOI:** 10.1371/journal.pone.0200765

**Published:** 2018-07-19

**Authors:** Hiran M. Thyagarajan, Jessica N. Lancaster, Sergio A. Lira, Lauren I. R. Ehrlich

**Affiliations:** 1 Department of Molecular Biosciences, Institute for Cellular and Molecular Biology, The University of Texas at Austin, Austin, Texas, United States of America; 2 Immunology Institute, Icahn School of Medicine at Mount Sinai, New York, New York, United States of America; 3 Livestrong Cancer Institutes, Dell Medical School, The University of Texas at Austin, Austin, Texas, United States of America; University of Alberta, CANADA

## Abstract

Following positive selection, thymocytes migrate into the medulla where they encounter diverse self-antigens that induce central tolerance. Thymocytes expressing T cell receptors (TCRs) with high affinity for self-antigens displayed by medullary antigen presenting cells (APCs) undergo either negative selection or diversion to the regulatory T cell (Treg) lineage, thus ensuring maturation of non-autoreactive T cells. Because many self-antigens are expressed by only a small percentage of medullary thymic epithelial cells, thymocytes must enter the medulla and efficiently scan APCs therein to encounter the full array of self-antigens that induce central tolerance. Chemokine receptors play a critical role in promoting medullary entry and rapid motility of post-positive selection thymocytes. We found that the chemokine receptor CCR8 is expressed by post-positive selection CD4^+^ single positive (SP) thymocytes in mice, while the corresponding chemokine ligands are expressed by medullary APCs, and thus hypothesized that CCR8 would promote thymocyte medullary entry and/or rapid motility to induce negative selection. However, despite a subtle decline in thymocyte medullary accumulation and the presence of autoantibodies in aged CCR8-deficient mice, CCR8 was not required for thymocyte differentiation, rapid motility, or negative selection.

## Introduction

Central tolerance in the thymus ensures that T cells that complete thymocyte maturation and selection are largely self-tolerant [1,2]. The thymus, which is divided into two main regions, the outer cortex and the central medulla, provides a specialized environment in which thymic stromal cells interact with thymocytes to induce their differentiation and selection [1,3]. As thymocytes mature, they must interact sequentially with cortical and then medullary stromal cells. Migration of thymocytes between the cortex and medulla is orchestrated by chemokine receptors, a subset of G-protein coupled receptors (GPCR) that are differentially expressed by distinct thymocyte maturation stages [4,5]. Thymic stromal cell subsets express different chemokines, creating spatial gradients that induce chemotactic migration of thymocytes through thymic compartments, where they interact with the relevant stromal subsets that induce their maturation and selection [2,4].

The chemokine receptors CCR7 and CCR9 promote entry of thymus-seeding progenitors into the thymus through blood vessels at the cortico-medullary junction (CMJ) [5,6]. Through interactions with cortical thymic epithelial cells (cTECs), partially mediated through CXCR4 signaling [7,8], these progenitors proliferate, commit to the T cell lineage, sequentially rearrange and express the genes encoding TCRβ and TCRα, and up-regulate expression of the co-receptors CD4 and CD8. Interactions between CD4^+^ CD8^+^ double positive (DP) thymocytes and cTECs induce positive selection of only those cells expressing TCRs with at least minimal affinity for self-peptide:major histocompatibility complex molecules (MHC)[1]. Thymocytes that pass the positive selection checkpoint survive, upregulate CD69, and migrate into the medulla, where they encounter self-antigens that are critical for establishing central tolerance.

Medullary thymic epithelial cells (mTECs) and dendritic cells (DCs) are the two predominant classes of medullary APCs that present diverse self-antigens to induce thymocyte self-tolerance against proteins expressed throughout the body [1]. Notably, the majority of the proteome is expressed by mTECs, partially due to their expression of the transcriptional regulator AIRE, which induces the ectopic expression of a large number of tissue restricted antigens (TRAs) that are otherwise expressed by only a few peripheral tissues [9–11]. However, individual TRAs are expressed by only 1–3% of mTECs, requiring thymocytes to scan a large number of mTECs to encounter the full array of self-antigens. Dendritic cells also display diverse self-peptides, some acquired from mTECs, some acquired from peripheral tissues, and some from the blood [12–14]. If a thymocyte expresses a TCR of sufficiently high affinity for self-antigens displayed by medullary APCs, it undergoes either negative selection (apoptosis) or is diverted to the Treg lineage. Deletion of even one medullary self-antigen in the thymus, can lead to spontaneous autoimmunity, highlighting the importance of medullary self-tolerance induction [15]. Thus, post-positive selection thymocytes must efficiently enter the medulla and scan the APCs therein, to encounter diverse antigens that induce complete central tolerance.

CCR7 is well established as a chemokine receptor that induces chemotaxis of post-positive selection CD4^+^ SP (CD4SP) and CD8^+^ SP (CD8SP) thymocytes towards the medulla and promotes negative selection therein [16–18]. However, we previously found that other chemokine receptors must contribute to this process [18]. We identified as candidates chemokine receptors that were expressed on thymocytes following positive selection, whose ligands were expressed by medullary stromal cells [19]. Both CCR4 and EBI2 fit this pattern, and we recently reported that these chemokine receptors contribute to thymocyte medullary entry and efficient negative selection [20,21]. We report here that the chemokine receptor CCR8 is also expressed by post-positive selection thymocytes, and its ligands CCL1 and CCL8 are expressed by medullary stromal cells.

CCR8 is expressed by Th2-polarized CD4^+^ T cells [22], CD4^+^ memory T cells in peripheral blood, and FOXP3^+^ Tregs in secondary lymphoid organs [23,24]. CCR8 promotes recruitment of Th2 cells to sites of allergic inflammation in the skin and lung, and may thus contribute to inflammation [24–27]. However, CCR8 is also expressed by peripheral Tregs and clearly plays a role in promoting their immunosuppressive activity, thus inhibiting autoimmunity [28–30]. The CCR8 ligand CCL8 recruits Th2 cells to sites of atopic skin inflammation [26], while the ligand CCL1 is implicated in promoting immunosuppressive activities of Tregs [30]. Furthermore, CCL1 has been shown to protect murine thymic lymphoma cell lines and thymocytes from dexamethasone mediated apoptosis *in vitro* [31,32]. With the exception of a study describing CD4^+^ T cell lineage-restricted expression of CCR8 [33], very little is known about the role of CCR8 in the thymus. Thus, we investigated the contribution of CCR8 to thymocyte medullary entry and negative selection.

Here, we demonstrate that CCR8 is expressed by post-positive selection CD4SP thymocytes while its ligands, CCL1 and CCL8 are expressed by mTECs and DCs in the thymic medulla. 2-photon imaging revealed that CCR8 deficiency resulted in a slight reduction in medullary accumulation of CD4SP thymocytes. However, CCR8 deficiency did not significantly alter thymocyte differentiation or selection. Thus, the presence of autoantibodies in the serum of aged CCR8-deficient mice, likely reflect a role for CCR8 in maintaining peripheral tolerance rather than establishing central tolerance.

## Materials and methods

### Mice

C57BL/6J (CD45.2), B6.SJL-Ptprc^a^ Pepc^b^ (CD45.1), B6.Cg-Tg(TcraTcrb)425Cbn/J (OT-II), and C57BL/6-Tg(Ins2-TFRC/OVA)296Wehi/WehiJ (RIP-mOVA) mice were purchased from The Jackson Laboratory. *Ccr8*^*-/-*^ and pCX-EGFP [18] strains were provided by Sergio A. Lira (Mount Sinai School of Medicine, NY) and Irving L. Weissman (Stanford University, Stanford, CA), respectively. OT-II *Ccr8*^*+/+*^, OT-II *Ccr8*^*-/-*^ and CD45.1/CD45.2 strains were bred in-house. Experiments were performed using mice 4–8 weeks of age of both genders, unless otherwise specified. All strains were bred and maintained under specific pathogen–free conditions at the University of Texas at Austin animal facility. Mouse maintenance and experimental procedures for this study were performed with approval from UT Austin’s Institutional Animal Care and Use Committee (IACUC) (protocol number AUP-2016-00101).

### Antibodies

For flow cytometric analyses of thymocyte and thymic stromal cell subsets the following fluorochrome- or biotin-conjugated antibodies were used (from eBioscience or BioLegend unless otherwise indicated): anti-CCR8-Alexa Fluor 647 (SA214G2; Biolegend), -CD8 (53–6.7), -CD69 (H1.2F3), -H-2K^b^ (AF6-88.5), -CD3 (145-2C11), -CD4 (RM4-5), -CD25 (PC61.5), -CD45.1 (A20), -CD45.2 (104), -Vα2 (B20.1), -Vβ5 (MR9-4), -CD11c (N418), -CD11b (M1/70), -B220 (RA3-6B2), -Gr-1 (RB6-8C5), -NK1.1 (PK136), -TER119 (TER-119), -cKit (2B8), -CD31 (390), -Sirp⍺ (P84), -I-A/I-E (M5/114.15.2), -CD80 (16-10A1), -CD45 (30-F11;BD Biosciences), -Ly51 (6C3), -EpCAM (G8.8), -Aire (5H12). Streptavidin Qdot^®^-605 (Life Technologies) was used to detect biotinylated antibodies.

For immunofluorescent analyses, the following antibodies were used: anti-keratin 5 (rabbit polyclonal; BioLegend), -pan-cytokeratin-FITC (C-11; Sigma Aldrich), -CD8-Alexa Fluor 594 (53–6.7; eBioscience), -CD4-APC (RM4-5; eBioscience), and donkey-anti-rabbit IgG conjugated to either DyLight 488 or DyLight 594 (polyclonal; Jackson ImmunoResearch Laboratories).

For enrichment of CD4 SP thymocytes for 2-photon imaging experiments, the following antibodies were used: anti-CD8(53.6.72; BioXCell), anti-CD25 (PC-61.5.3; BioXCell), anti-B220 (clone RA3.3A1/6.1; BioXCell), anti-Ter119 (BE0183; BioXCell), anti-Gr1 (RB6-8C5; BioXCell), anti-CD11b (M1/70; BioXCell). T cell depletion for bone marrow chimeras was done using anti-CD3 (17A2; BioXCell).

### Flow cytometric analysis

Single-cell suspensions of thymocytes were obtained by manually dissociating thymic tissue, and filtering cells through 40μm strainers (Thermo Fisher Scientific). 5 x 10^6^ thymocytes were incubated with a cocktail of fluorochrome-conjugated or biotinylated antibodies for 20 min in the dark at 4°C in FACS wash (FW; PBS+ 2% bovine calf serum; GemCell), followed by incubation with Streptavidin Qdot 605 (Life Technologies), prior to resuspension in FW with 0.1 μg/mL Propidium iodide (PI; Enzo Life Sciences) to distinguish live/dead cells. Samples were analyzed using an LSR Fortessa flow cytometer (BD Biosciences); FlowJo ver.9.9.5 (Tree Star) was used for data analysis. For CCR8 surface detection, cells were first immunostained with anti-CCR8-Alexa Fluor 647 (SA214G2; Biolegend) for 30 minutes at room temperature, followed by immunostaining for other cell-surface proteins as described above. To analyze DNA content, cells were immunostained with antibodies against surface markers, then permeabilized with 70% ethanol, and stained with 1μg/ml PI in PBS.

### cDNA preparation and qRT-PCR

cDNA preparation and qRT-PCR of sorted thymocyte subsets was done as previously described [20]. Briefly, thymocytes isolated from 1-month old C57BL/6J mice were immunostained with fluorophore-conjugated antibodies against CD8, CD69, CD3, CD4, CD25, CD44, CD11c, CD11b, B220, Gr-1, NK1.1, TER119, and cKit, and thymocyte subsets were sorted to >95% purity on a FACS Aria II (BD Biosciences). cDNA preparation and qRT-PCR of sorted thymic stromal cells was performed as previously described [21]. In brief, thymi were enzymatically dissociated using a cocktail of Liberase TM (Roche) and DNase I (Roche). Stromal cells were then immunostained with FITC-labeled Ulex europaeus agglutinin 1 (UEA-1; Vector Laboratories) and fluorophore-conjugated antibodies against I-A/I-E, CD45, TER-119, EpCAM, CD11c, Ly-51, CD80, Sirp-α and B220. Stromal subsets were sorted to >95% purity on a FACSAria II (BD Biosciences), as described previously [20,21]. Sorted cells were resuspended in TRIzol (Life Technologies), RNA was extracted, and cDNA was generated using SuperScript^®^ III First-Strand Synthesis SuperMix (Life Technologies). qRT-PCR was performed as described [20] using the SYBR Green Real-Time PCR mastermix on a ViiA 7 Real-Time PCR system using the following primers: CCR8 forward ACGTCACGATGACCGACTACT, CCR8 reverse CCCAGCACAAACAAGACGC, CCL1 forward GGCTGCCGTGTGGATACAG, CCL1 reverse AGGTGATTTTGAACCCACGTTT, CCL8 forward CTGGGCCAGATAAGGCTCC, and CCL8 reverse CATGGGGCACTGGATATTGTT.

### Immunofluorescence analysis of thymic cryosections and detection of autoantibodies in mouse serum

Thymi from 5–7 week-old *Ccr8*^*+/+*^ and *Ccr8*^*-/-*^ mice were embedded in Tissue-Tek OCT (Sakura) and snap frozen. 7 μm cryosections were generated with a Microm HM550 Cryostat (ThermoFisher) and stored at -80°C. Prior to immunostaining, sections were fixed in 100% acetone at -20°C for 20 minutes and washed with PBS + 0.1% Tween20. Immunostaining was carried out for 1h at 4°C with the following primary antibodies: anti-pancytokeratin-FITC, -keratin 5, -CD4-APC and -CD8-Alexa Fluor 594. Donkey anti-rabbit IgG-DyLight 594 secondary reagent was used to detect the anti-keratin 5 antibody. 4’,6-diamidino-2-phenylindole (DAPI; Life technologies) was used at 0.125μg/ml in PBS to detect nuclei.

To detect the presence of anti-nuclear autoantibodies in mouse serum, 7 μm kidney cryosections from *Rag2*^*-/-*^ mice were immunostained. The cryosections were first fixed in 100% acetone as described above, followed by overnight incubation with undiluted mouse serum from *Ccr8*^*+/+*^, *Ccr8*^*-/-*^ and *Ccr7*^*-/-*^ mice at 4°C. Slides were washed with PBS + 0.1% Tween 20, then incubated with donkey anti-mouse IgG-Alexa Fluor 594 to detect murine autoantibodies, and then stained with DAPI.

All immunostained sections were mounted using ProLong Gold antifade reagent (Life Technologies) and imaged on a Leica DMi8 microscope with 10x/0.4 NA and 20x/0.7 NA objectives, using LasX software. Images were processed uniformly using FIJI software v2.0.0.

### Analysis of cortical and medullary thymic areas

Quantification of cortical and medullary areas from immunofluorescent slide images was done using an automated MATLAB (Mathworks, Natick, MA) code to calculate the areas of medullary regions, cortical regions, and overlap as previously described [34]. Briefly, the two color channels were first identified, and two new regions were created to determine non-overlapping regions for strictly medullary or strictly cortical areas. Following this, color thresholds were applied to cortical and medullary regions and the two regions corresponding to cortex and medulla were morphologically closed to preserve the image shape. The overlap areas were determined by comparing the complete masks for the two color channels and identifying co-stained pixels. Following identification of the regions of interest (total, overlap, medullary only, and cortical only) composite images made, with each region processed to identify the number of distinct regions and their corresponding pixel sizes. The physical areas were calculated using image pixel dimensions unique to the imaging system. Automated MATLAB code is available upon request.

### Generation and analysis of bone marrow chimeras

For competitive bone marrow chimeras, bone marrow (BM) was extracted from femurs of CD45.1, *Ccr8*^*+/+*^ CD45.2 and *Ccr8*^*-/-*^ CD45.2 mice. T cells were depleted by first immunostaining for 30 minutes with anti-CD3(BioXCell) (17A2) at 4°C, followed by magnetic depletion with anti-rat IgG Dynabeads^®^ (Invitrogen). 5 x 10^6^ CD45.1 cells were mixed with an equal number of CD45.2 *Ccr8*^*+/+*^ or CD45.2 *Ccr8*^*-/-*^ cells and transplanted, via retro-orbital injection, into lethally irradiated (900 rad delivered in split doses) CD45.1/CD45.2 recipient mice, 6–8 weeks of age. 6 weeks after reconstitution, thymic chimerism was assessed by flow cytometry. For OT-II chimeras, donor bone marrow from *Ccr8*^*+/+*^ OT-II or from *Ccr8*^-/-^OT-II mice was transplanted into lethally irradiated RIP-mOVA^+^ or RIP-mOVA^-^ recipient mice as indicated.

### Cell viability assay

10^6^ thymocytes from *Ccr8*^+/+^or *Ccr8*^-/-^ mice were incubated at 37°C, 5% CO_2_ for 24 hours in 200μl of complete RPMI (RPMI-1640 medium [Gibco] + 10% FBS [Hyclone], 1x GlutaMAX, 1x Penicillin [100U/ml]- Streptomycin [100μg/ml]-Glutamine [300μg/ml], 1mM Sodium Pyruvate, 1x MEM NEAA, and 50μM 2-mercaptoethanol [Gibco]), with or without CCL8. After 24 hours, cells were immunostained with AnnexinV-PE (eBiosicence) and resuspended in FW + 0.1μg/ml PI (Enzo), and viability (AnnexinV^-^PI^-^) was assessed by flow cytometry.

### Two-photon imaging

CD4SP thymocytes were enriched via magnetic depletion using anti-rat Dynal beads (Life Technologies) following incubation with antibodies against CD8, Gr-1, Ter119, B220, CD25, and CD11b (BioXCell). Flow cytometry was used to confirm > 90% purity of enriched CD4SP thymocytes. 1x10^6^ each *Ccr8*^*+/+*^ and *Ccr8*^*-/-*^ CD4SP cells were stained with Indo-1AM (Sigma) and CMTPX red (Life Technologies), respectively, for 30 minutes at 37°C, according to manufacturers’ instructions, then mixed at a 1:1 ratio in complete RPMI. The CellTracker dyes for *Ccr8*^*+/+*^ and *Ccr8*^*-/-*^ CD4SP cells were switched between independent repeats to eliminate color effects. 3–4 week old pCX-EGFP thymi were embedded in NuSieve GTG low-melt agarose (Lonza), then sectioned with a VT1000S Vibratome (Leica), as previously described [35]. The enriched CD4SP T cells were applied to the thymic slices, which were imaged by two-photon microscopy following a 1–2 hour incubation period at 37°C, 5% CO_2_. Images were acquired every 15 seconds through a depth of 40 μm, at 5 μm intervals, using an Ultima IV microscope equipped with a 20X NA 1.0 water-immersion objective (Olympus), and PraireView software v5.3 (Prairie). Mai-Tai 2-photon lasers (SpectraPhysics) tuned to 740 nm and 900 nm were used to excite Indo-1 and EGFP/CMTPX, respectively, and the emitted light was detected using 400/50, 480/40, 535/50, and 607/45 filters (Chroma Technology) for detection of the two Indo-1 emission peaks, EGFP, and CMTPX, respectively. Cell tracking and analysis were carried out with Imaris image analysis software v.9.1 (Bitplane). Path straightness was calculated as the ratio of cell displacement over total path length.

### Statistical analysis

Statistical analysis wherever indicated in the figure legends were performed using Prism 6 (GraphPad). P-values are marked with asterisks (*); * *p* < 0.05; ** *p* < 0.01; *** *p* < 0.001; **** *p* < 0.0001.

## Results

### CCR8 is expressed by post-positive selection thymocytes, while its ligands are expressed by medullary thymic stromal cell subsets

**Fig 1:**
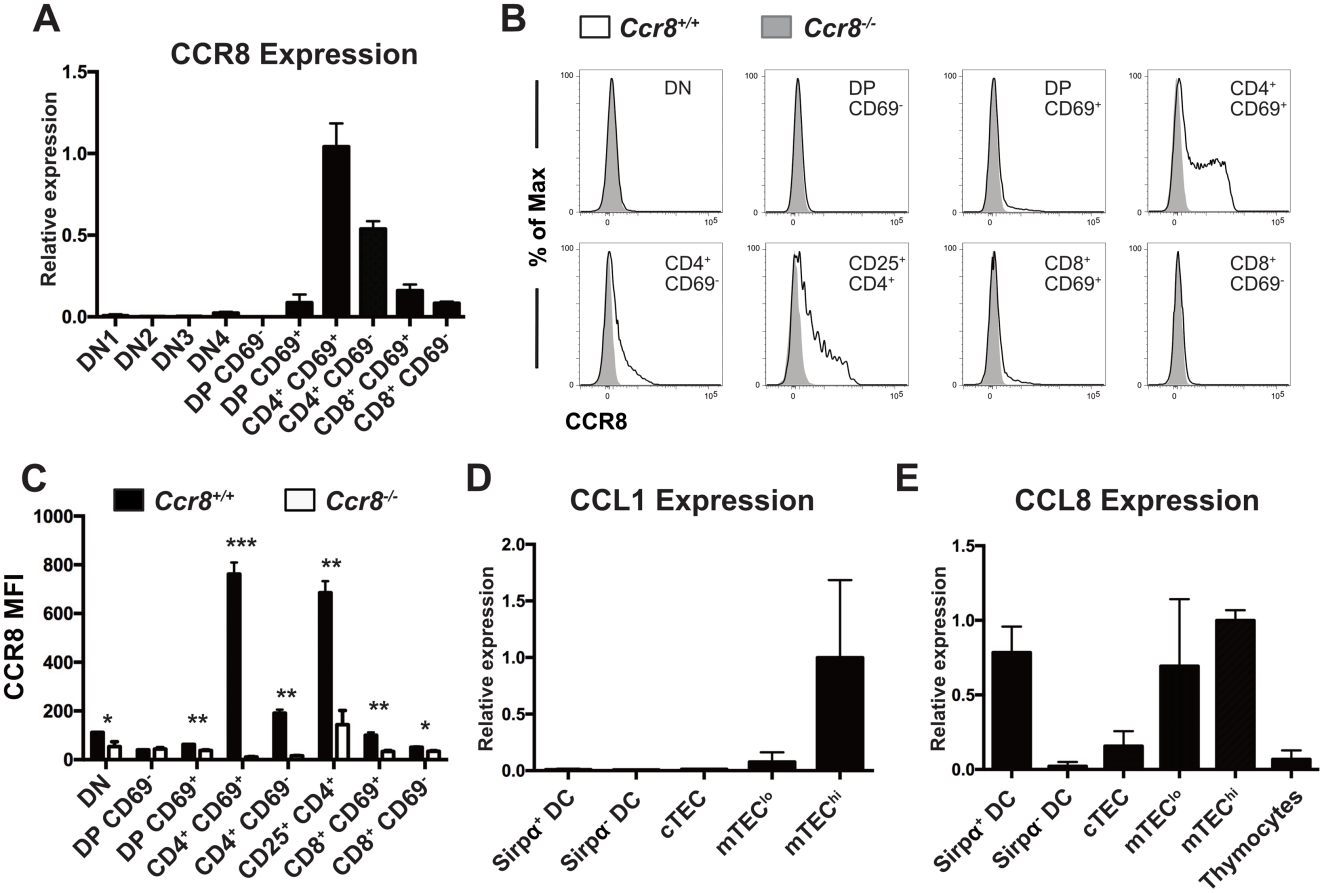
CCR8 is expressed by post-positive selection thymocytes, and CCR8 ligands are expressed by medullary thymic stromal cell subsets. (A) qRT-PCR analysis was used to quantify relative *Ccr8* expression levels in FACS-sorted thymocyte subsets. Expression was normalized first to β-actin levels in each sample, and then between samples to expression in the CD4^+^ CD69^+^ subset. Graph shows means + SD of technical triplicates from one representative of three independent experiments. (B) Flow cytometric profiles of CCR8 cell surface expression on thymocyte subsets from *Ccr8*^*+/+*^ and *Ccr8*^*-/-*^ mice. Data are representative of two independent experiments, with a total of six mice per genotype. (C) Quantification of CCR8 Mean Fluorescence Intensities (MFI) in *Ccr8*^*+/+*^ to *Ccr8*^*-/-*^ thymocyte subsets, from data as in (B). Graph shows means + SD from one representative experiment with 3 mice per group; *p < 0.05, **p < 0.01, ***p < 0.001 (Unpaired two-tailed *t-test* with the Holm-Sidak multiple comparisons testing). (D and E) qRT-PCR analysis was used to quantify relative mRNA expression levels of the CCR8 ligands CCL1 and CCL8 in the indicated FACS-sorted thymic stromal cell subsets. Expression was normalized first to β-actin levels in each sample, and then between samples to expression in the mTEC^hi^ subset. Graph shows means + SEM compiled from two independent experiments, each with three technical replicates.

Analysis of our previous gene expression profiling data from thymocyte and thymic stromal cell subsets revealed that CCR8 was highly expressed by early post-positive selection CD4SP cells, and to some extent by CD8SP cells, while the ligand CCL1 was expressed by MHCII^hi^CD80^hi^ mTEC (mTEC^hi^) cells, and the ligand CCL8 was expressed by both mTECs and DCs [19]. qRT-PCR analysis of FACS sorted thymocyte subsets (Figure A in S1 Fig) confirmed that post-positive selection CD4^+^ CD69^+^ thymocytes upregulated CCR8 expression, which was diminished in the mature CD4^+^ CD69^-^ cells; CD8SP cells expressed minimal levels of CCR8 (Fig 1A). This expression pattern was confirmed at the protein level by flow cytometry (Fig 1B and 1C) and is largely consistent with a previous report [33]. CCR8 was also expressed by CD4^+^ CD25^+^ thymocytes, which consists of Treg and Treg progenitor subsets. Further delineation of CD4SP cells into maturation subsets defined by expression of CD69 and MHCI (Figure A in S2 Fig) [36] revealed that CCR8 was expressed by some CD69^+^MHCI^-^ (SM) and CD69^+^MHCI^+^ (M1) CD4SP cells. Heterogeneous CCR8 expression in SM and M1 subsets could reflect either maturation state or downregulation of the cell surface receptor upon signaling. In contrast to CCR4, which is expressed at the highest levels by post-positive selection DP and immature CD4SP SM cells [20], CCR8 is expressed mainly by the more mature M1 CD4SP subset, suggesting it could have a distinct role in thymocyte selection.

Expression of the two known CCR8 chemokine ligands, CCL1 and CCL8, was also confirmed by qRT-PCR analysis of FACS purified thymic stromal cell subsets (Figure B in S1 Fig). CCL1 was expressed by mTEC^hi^ cells (Fig 1D), while CCL8 was expressed by both mTECs and the Sirpα^+^ subset of conventional thymic DCs [37] (Fig 1E). Both mTECs and Sirpα^+^ DC subsets are located in the medulla and play a crucial role in establishing central tolerance through the presentation of self-antigens to maturing SP thymocytes [2]. Thus, the expression pattern of CCR8 and its ligands is consistent with a potential role for CCR8 in thymocyte medullary entry and negative selection.

### CCR8 deficiency does not significantly alter thymic architecture

**Fig 2:**
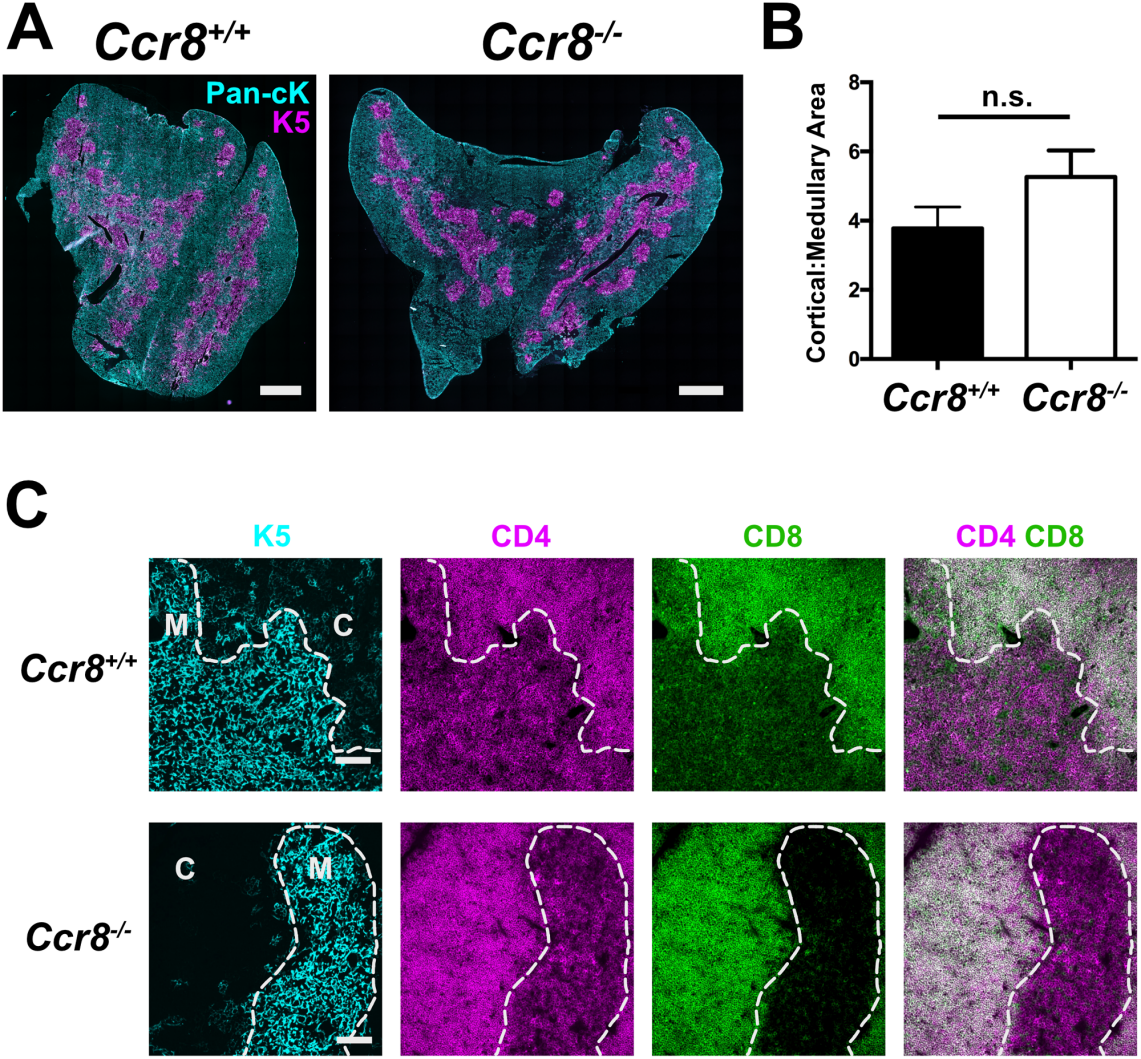
CCR8 deficiency does not grossly perturb thymic architecture or alter thymocyte subset localization. (A) Representative immunostaining of *Ccr8*^*+/+*^ and *Ccr8*^*-/-*^ thymic cryosections with antibodies against pan-cytoKeratin (Pan-cK; cyan) and Keratin 5 (K5; magenta), to reveal cortical and medullary epithelium, respectively. Bar 1mm. (B) Ratios of cortical to medullary areas in *Ccr8*^*+/+*^ and *Ccr8*^*-/-*^ thymic cryosections were calculated from immunofluorescence images as in (A). Mean + SEM are shown for 5 *Ccr8*^*+/+*^ mice and 7 *Ccr8*^*-/-*^ mice; n.s. not significant (Unpaired Student’s *t* test). (C) Immunostaining of *Ccr8*^*+/+*^ and *Ccr8*^*-/-*^ thymic cryosections with antibodies against keratin 5 (K5; cyan), to mark medullary epithelial cells, as well as CD4 (magenta) and CD8 (green) to distinguish DP, CD4SP, and CD8SP thymocyte subsets. The white dashed line separates cortex (C) from medulla (M). Bar 100μm. Representative of three mice per group.

Thymocyte: stromal cell crosstalk is crucial for proper differentiation of both thymocytes and thymic stromal cells. mTEC proliferation and maturation is driven by interactions with SP thymocytes expressing RANKL and CD40L [38–40]. Thus, because CCR7-deficient SP thymocytes do not enter the medulla to interact efficiently with mTECs and drive their maturation [18], *Ccr7*^-/-^ thymi are characterized by smaller, spatially distributed medullary regions [18,41]. We reasoned that if CCR8 deficiency impaired thymocyte medullary entry, the cortical: medullary organization of *Ccr8*^*-/-*^ thymi would be similarly impaired. However, quantitative analysis of immunofluorescence images did not reveal significant disorganization of cortical or medullary areas in *Ccr8*^-/-^ thymi (Fig 2A and 2B) [34]. Furthermore, DP cells were properly localized to the cortex of *Ccr8*^-/-^ thymi at steady state, and CD4SP and CD8SP cells were localized to the medulla (Fig 2C). Thus, CCR8 deficiency does not significantly disrupt thymic architecture or steady-state intrathymic localization of thymocyte subsets.

### CCR8 is not required for negative selection of polyclonal thymocytes

**Fig 3:**
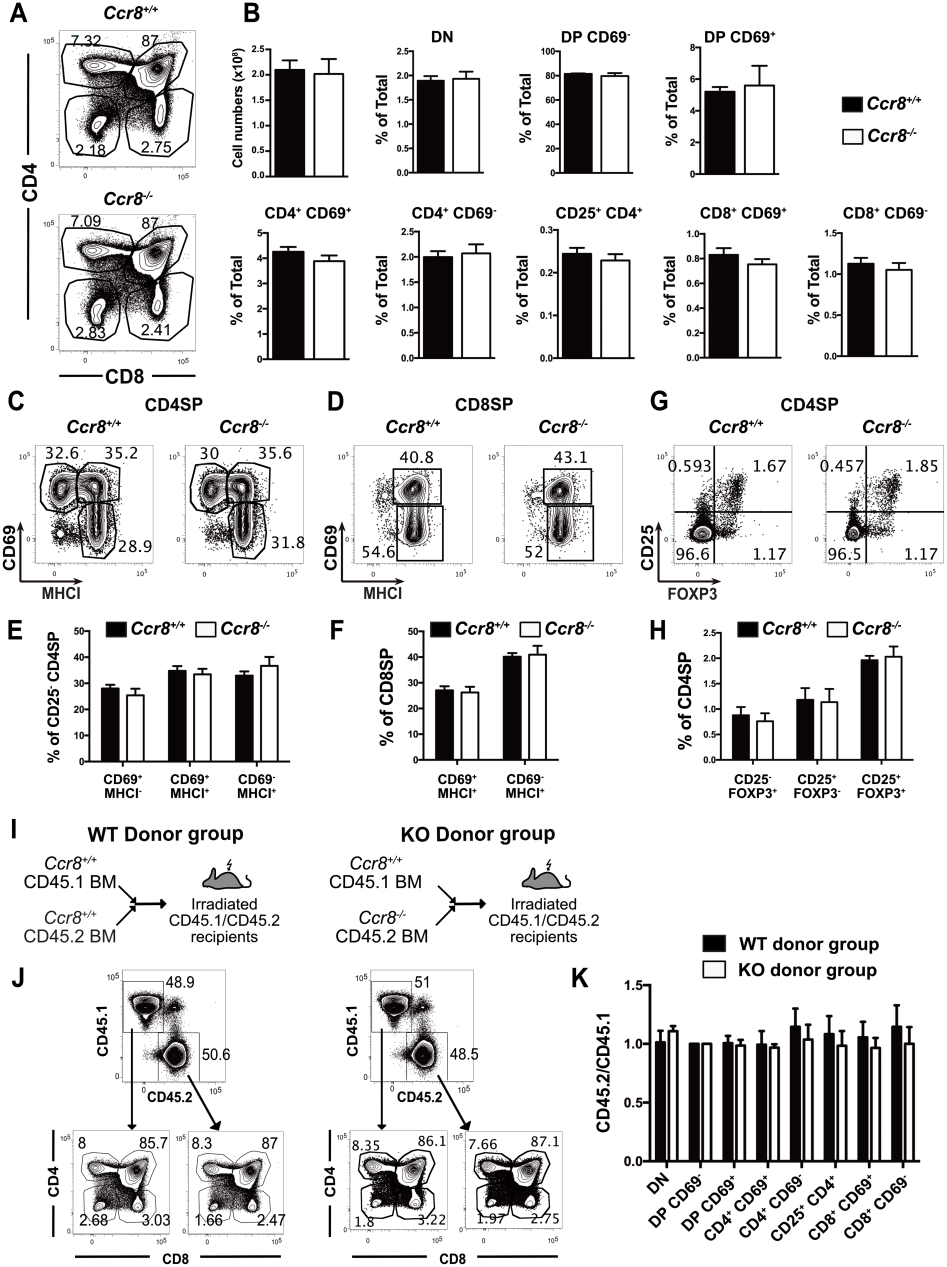
CCR8 is not required for negative selection of polyclonal thymocytes. (A) Representative flow cytometric profiles of CD4 versus CD8 expression on thymocytes from *Ccr8*^*+/+*^ and *Ccr8*^*-/-*^ mice. (B) Total thymocyte cellularity was quantified, and the percentages of thymocyte subsets in *Ccr8*^*+/+*^ and *Ccr8*^*-/-*^ mice were calculated from flow cytometric data, as in (A). Graphs show means + SEM compiled from four independent experiments with a total of 12 *Ccr8*^*+/+*^ and 11 *Ccr8*^*-/-*^ mice. (C-D) Representative flow cytometric profiles of CD69 versus MHCI expression on gated CD4SP (C) and CD8SP (D) thymocytes from *Ccr8*^*+/+*^ and *Ccr8*^*-/-*^ mice. (E-F) The percentage of each indicated maturation stage within CD25^-^ CD4SP (E) or CD8SP (F) subsets were quantified based on flow cytometric data as in (C-D). Graphs show means + SEM compiled from two independent experiments with a total of 6 *Ccr8*^*+/+*^ and 5 *Ccr8*^*-/-*^ mice. (G-H) Representative flow cytometric profiles of CD25 versus FOXP3 expression on CD4SP thymocytes (G), and quantification of the frequency of Treg and Treg progenitors defined by these gates (H). Graphs depict means + SEM compiled from three independent experiments with a total of 9 *Ccr8*^*+/+*^ and 9 *Ccr8*^*-/-*^ mice. (I) Experimental schematic for generation of mixed bone marrow chimeras in which *Ccr8*^*+/+*^ CD45.1 bone marrow was mixed 1:1 with either *Ccr8*^*+/+*^ CD45.2 or *Ccr8*^*-/-*^ CD45.2 bone marrow prior to transplantation into lethally irradiated congenic CD45.1/CD45.2 recipients. (J) Representative flow cytometry plots of CD45.1 versus CD45.2 profiles and downstream CD4 versus CD8 profiles from analysis of mixed bone marrow chimera recipients analyzed six weeks after reconstitution. (K) The ratio of CD45.2 to CD45.1 donor chimerism was calculated for the indicated thymocyte subsets from flow cytometric analysis of the mixed bone marrow chimera recipients as in (J), and the values were normalized to the DP CD69^-^ subset. Graphs show means + SEM compiled from three independent experiments with a total of 9 WT donor group recipients and 10 KO donor group recipients.

To determine whether CCR8 deficiency impacts thymocyte differentiation, we quantified the cellularity of thymocyte subsets in *Ccr8*^*-/-*^ versus *Ccr8*^*+/+*^ mice by flow cytometry. CCR8 deficiency did not alter total thymocyte cellularity or the relative proportions of the predominant thymocyte subsets (Fig 3A and 3B). CCR8 deficiency also did not significantly alter progression through SP maturation stages, as delineated by expression of CD69 and MHCI [36], in the CD4SP or CD8SP compartments (Fig 3C–3F). Furthermore, CCR8 deficiency did not alter the frequencies of FOXP3^+^CD25^+^ Treg or their intrathymic FOXP3^+^CD25^-^ and FOXP3^-^CD25^+^ precursor populations [42] (Fig 3G and 3H) within the CD4SP compartment. Given the comparable number of CD4SP cells, these data indicate that CCR8 is not required for Treg generation. Thus, CCR8 deficiency did not significantly alter any aspect of polyclonal thymocyte differentiation examined.

Subtle changes in thymocyte differentiation arising from CCR8 deficiency could potentially be compensated for by homeostatic mechanisms and might become more apparent in a competitive setting. For example, CCR4 deficiency does not alter thymocyte cellularity or composition at steady state, but in a competitive bone marrow chimera setting, CCR4-deficient thymocytes were overrepresented compared to wild-type cells at all post-positive selection thymocyte stages, reflecting impaired negative selection of CCR4-deficient thymocytes [20]. Thus, we generated mixed bone marrow chimeras by transplanting a 1:1 mixture of T cell-depleted bone marrow cells from *Ccr8*^*+/+*^ CD45.1 mice with either *Ccr8*^*+/+*^ CD45.2 or *Ccr8*^*-/-*^ CD45.2 cells into lethally irradiated CD45.1/CD45.2 recipients (Fig 3I). Six weeks after reconstitution, thymocyte chimerism was assessed by flow cytometry (Fig 3J). The relative frequencies of thymocyte subsets from each congenic donor were calculated and normalized to the DP CD69^-^ subset, in which *Ccr8* is not yet expressed. Thymocytes derived from CCR8 deficient donors were not over- or under-represented relative to those from wild-type donors at any stage of thymocyte differentiation (Fig 3K). Therefore, even in a competitive environment, CCR8 deficiency did not alter thymocyte differentiation.

### CCR8 is dispensable for selection of monoclonal CD4SP thymocytes responding to a model medullary TRA

We next assessed the impact of CCR8 deficiency on the ability of thymocytes of a known specificity to undergo negative selection in response to their cognate ligand. OT-II TCR transgenic thymocytes express an MHCII-restricted TCR with specificity for a peptide of ovalbumin (OVA) presented by I-A^b^ [43]. To induce negative selection, we used RIP-mOVA transgenic mice, in which a transmembrane form of OVA is expressed by mTEC cells under control of the rat insulin promoter [44]. Negative selection of OT-II thymocytes in RIP-mOVA mice is *Aire* dependent, and RIP-mOVA serves as a model TRA for the OT-II TCR [45]. We confirmed that CCR8 was expressed, although at a lower level, by comparable CD4SP subsets in OT-II versus polyclonal thymocytes (Figure B in S2 Fig).

**Fig 4:**
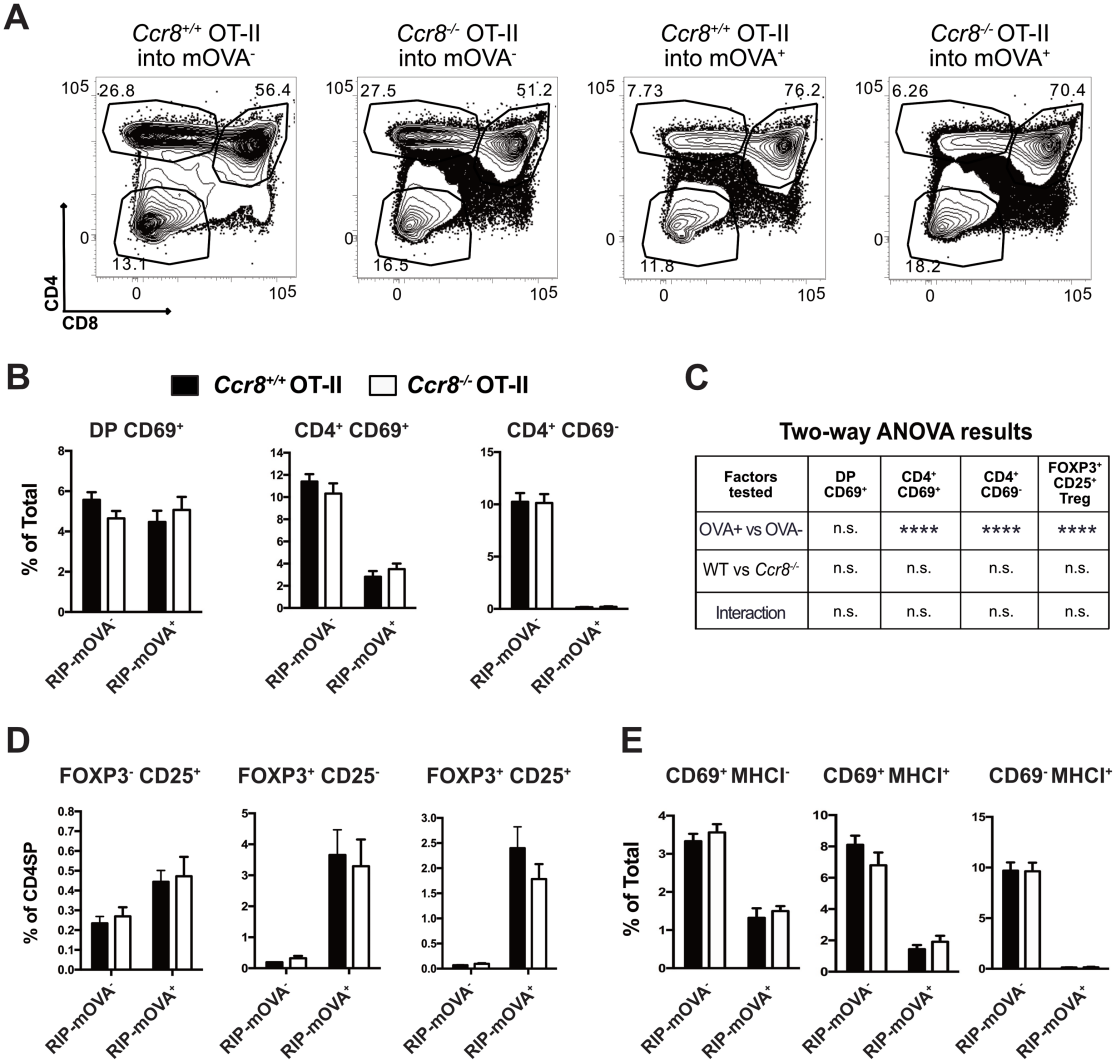
CCR8 is dispensable for negative selection, Treg induction, and differentiation of monoclonal CD4SP thymocytes responding to a model TRA. (A) Representative CD4 versus CD8 flow cytometric profiles of thymocytes analyzed after transplantation of *Ccr8*^+/+^ OT-II or *Ccr8*^-/-^ OT-II bone marrow progenitors into RIP-mOVA^+^ or RIP-mOVA^-^ recipients, as indicated. Thymocyte chimerism was analyzed 6 weeks after transplantation. (B-E) Analysis of OT-II chimeras shown in (A). (B) The percentages of *Ccr8*^+/+^ and *Ccr8*^-/-^ OT-II thymocyte subsets were quantified based on flow cytometric analysis. (C) Two-way ANOVA was used to determine whether the frequency of the indicated thymocyte subsets was significantly impacted by the CCR8 genotype, the presence of OVA, or the interaction of these two factors in the OT-II bone marrow chimeras. (D) Quantification of the percent of each Treg or Treg precursor subset within CD3^+^ CD4SP compartment, as assessed by flow cytometry. (E) The frequencies of OT-II CD4SP maturation subsets defined by CD69 and MHCI expression were determined by flow cytometry. All graphs depict means + SEM compiled from two independent experiments with a total of n = 6 OT-II *Ccr8*^*+/+*^ → RIP mOVA^-^; n = 5 OT-II *Ccr8*^*+/+*^ → RIP mOVA^+^; n = 6 OT-II *Ccr8*^*-/-*^ → RIP mOVA^-^; n = 6 OT-II *Ccr8*^*-/-*^ → RIP mOVA^+^ bone marrow chimera recipients.

To determine whether CR8 deficiency impaired RIP-mOVA-mediated negative selection of OT-II thymocytes, we transplanted T cell-depleted OT-II bone marrow sufficient or deficient for CCR8 into lethally irradiated RIP-mOVA^+^ or RIP-mOVA^-^ recipients. Thymocyte chimerism was analyzed by flow cytometry six weeks after transplantation (Fig 4A). Negative selection of *Ccr8*^+/+^ CD4SP thymocyte subsets occurred as expected in RIP-mOVA recipients; however, CCR8 deficiency did not impair negative selection of OT-II CD4SP cells in response to the RIP-mOVA TRA (Fig 4B). Two-way ANOVA analysis was used to test for statistical significance of the presence of OVA, the genotype of CCR8, or the interaction of these factors on the frequency of post-positive selection OT-II thymocytes subsets (Fig 4C). While OVA induced significant deletion of CD4SP subsets, neither CCR8 deficiency nor the interaction of OVA with CCR8 deficiency significantly impacted the frequency of these thymocyte subsets. While RIP-mOVA induced significant generation of OT-II Treg, CCR8 deficiency did not alter the frequencies of FOXP3^+^ Treg or Treg precursor subsets within the CD4SP lineage (Fig 4C and 4D). Analysis of CD69 versus MHCI expression by OT-II CD4SP thymocytes, also showed that CCR8 deficiency did not impact maturation of OT-II CD4SP thymocytes in the presence or absence of the RIP-mOVA TRA (Fig 4E). These data indicate that CCR8 is not required for negative selection, Treg induction, or maturation of CD4SP thymocytes.

The cellularity of all conventional thymocyte subsets was increased in recipients repopulated with CCR8 deficient OT-II bone marrow in the chimeras shown in Fig 4 (Figures A and B in S3 Fig), although thymocyte cellularity was not increased in polyclonal CCR8-deficient mice (Fig 3B). This increased cellularity did not reflect a change in the frequencies of DN, DP or SP thymocyte subsets between CCR8-deficient and CCR8-sufficient recipients (Fig 4B and 4C and Figure C in S3 Fig), indicating thymocyte maturation as intact. To further assess whether CCR8 deficiency impacted negative selection of OT-II thymocytes, we calculated the percent deletion of *Ccr8*^*+/+*^ and *Ccr8*^*-/-*^ OT-II thymocyte subsets induced by the presence of OVA in these bone marrow chimera recipients. This analysis confirmed that CCR8 deficiency did not impact negative selection of OT-II thymocytes (Figure D in S3 Fig). The increased thymocyte cellularity in CCR8-deficient OT-II bone marrow recipients could reflect a role for CCR8 in regulating overall thymocyte proliferation or survival. However, CCR8 deficiency did not alter the frequency of DN, DP, or CD4SP thymocyte subsets undergoing proliferation in OT-II bone marrow chimeras (Figures E and F in S3 Fig). To test the impact of CCR8 deficiency on thymocyte apoptosis independent of TCR stimulation, we cultured *Ccr8*^*+/+*^ OT-II and *Ccr8*^*-/-*^ OT-II thymocytes overnight in the presence or absence of CCL8, and subsequently assessed viability by flow cytometry. CCR8 deficiency did not result in increased survival of OT-II CD4SP subsets (Figure G in S3 Fig), or any other thymocyte subset (not shown). Addition of CCR8 ligands also did not impact survival of CCR8-sufficient CD4SP cells (Figure G in S3 Fig). Thus, it remains unclear why CCR8 deficiency in the OT-II bone marrow chimera setting resulted in increased thymocyte cellularity, but we found no evidence that CCR8 impacted OT-II thymocyte differentiation, selection, survival, or proliferation.

### CCR8 promotes medullary enrichment of CD4SP thymocytes

**Fig 5:**
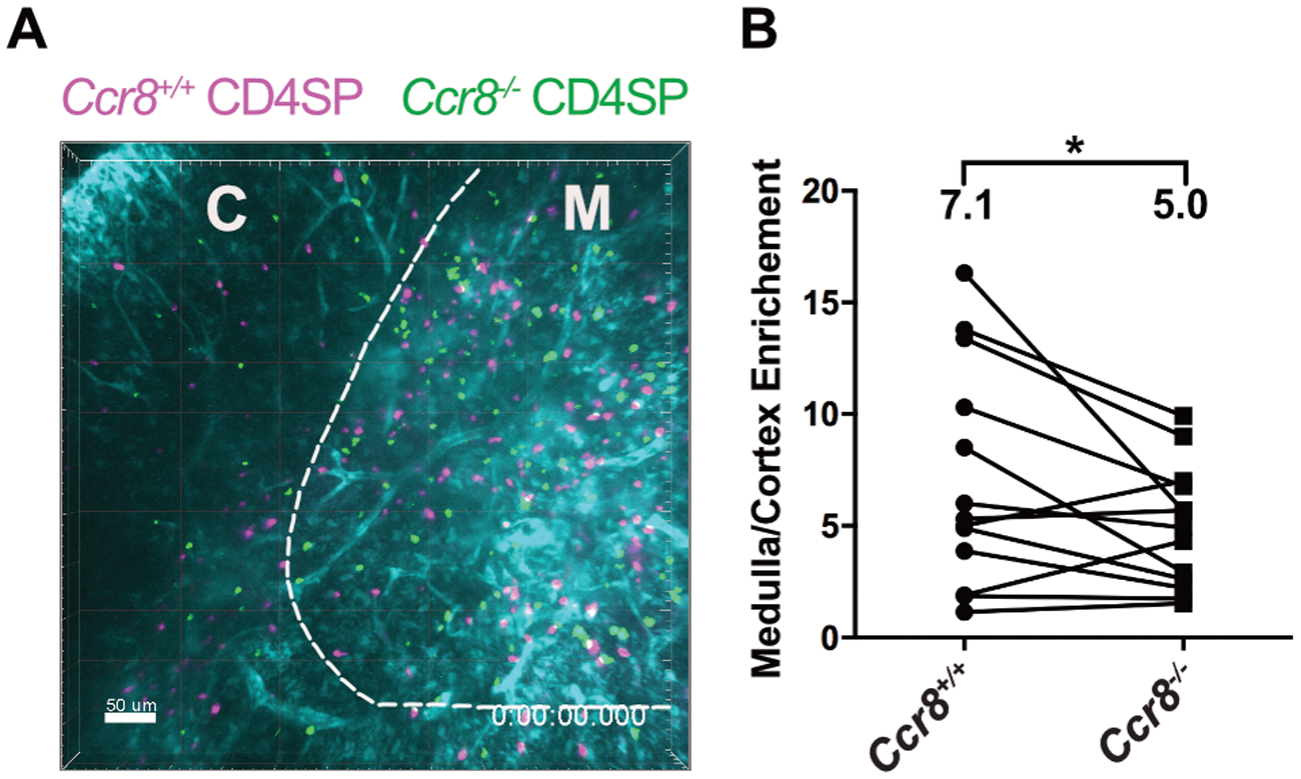
CCR8 promotes medullary enrichment of CD4SP thymocytes. (A) A maximum intensity projection of *Ccr8*^*+/+*^ (pink) and *Ccr8*^*-/-*^ (green) CD4SP thymocytes in live pCX-EGFP (cyan) thymic slices, from 2-photon fluorescence microscopy images. The boundary between cortex (C) and medulla (M) is indicated with a dashed line. Bar 50μm; acquired with a 20X objective. See also S1 Movie. (B) The density of cells (cells/μm^2^) in the medulla and in the cortex was calculated from images as in (A), and enrichment of CD4SP cells in the medulla was determined as a ratio of medullary to cortical densities. CD4SP medullary enrichment was calculated from three independent imaging experiments, with a total of 11 slices analyzed. *p < 0.05 (paired Student’s *t*-test).

We initially hypothesized that CCR8 expression by CD4SP cells would promote chemotaxis towards the CCR8 ligands expressed by medullary thymic stromal subsets, contributing to accumulation of CD4SP cells in the medulla. To test this hypothesis, we employed a 2-photon imaging approach to visualize the motility and localization of *Ccr8*^*+/+*^ and *Ccr8*^*-/-*^ CD4SP thymocytes in live thymic slices. Thymic slices were generated from pCX-EGFP transgenic mice, in which cortical and medullary regions can be distinguished based on the GFP intensity and morphology of GFP^+^ cells. We previously used this system to show that CD4SP thymocytes accumulated at a higher density in the medulla relative to the cortex in a GPCR-dependent manner [18]. Consistent with our hypothesis, CCR8 deficiency resulted in a slight, but significant, decrease in the accumulation of CD4SP thymocytes in the medulla (Fig 5A and 5B). The fact that this decrease is slight likely reflects the role of other chemokine receptors, such as CCR7 and CCR4, in promoting CD4SP medullary entry [18,20]. Also, the impact of CCR8 expression on medullary enrichment may underestimated given that only 35% of CD4SP cells express CCR8 (Figure A in S2 Fig). CCR8 deficiency did not alter the velocity or path straightness of migrating CD4SP thymocytes (Figures A and B in S4 Fig). These data indicate a minor role for CCR8 in promoting CD4SP localization to the medulla.

### CCR8 deficiency results in increased production of autoantibodies

**Fig 6:**
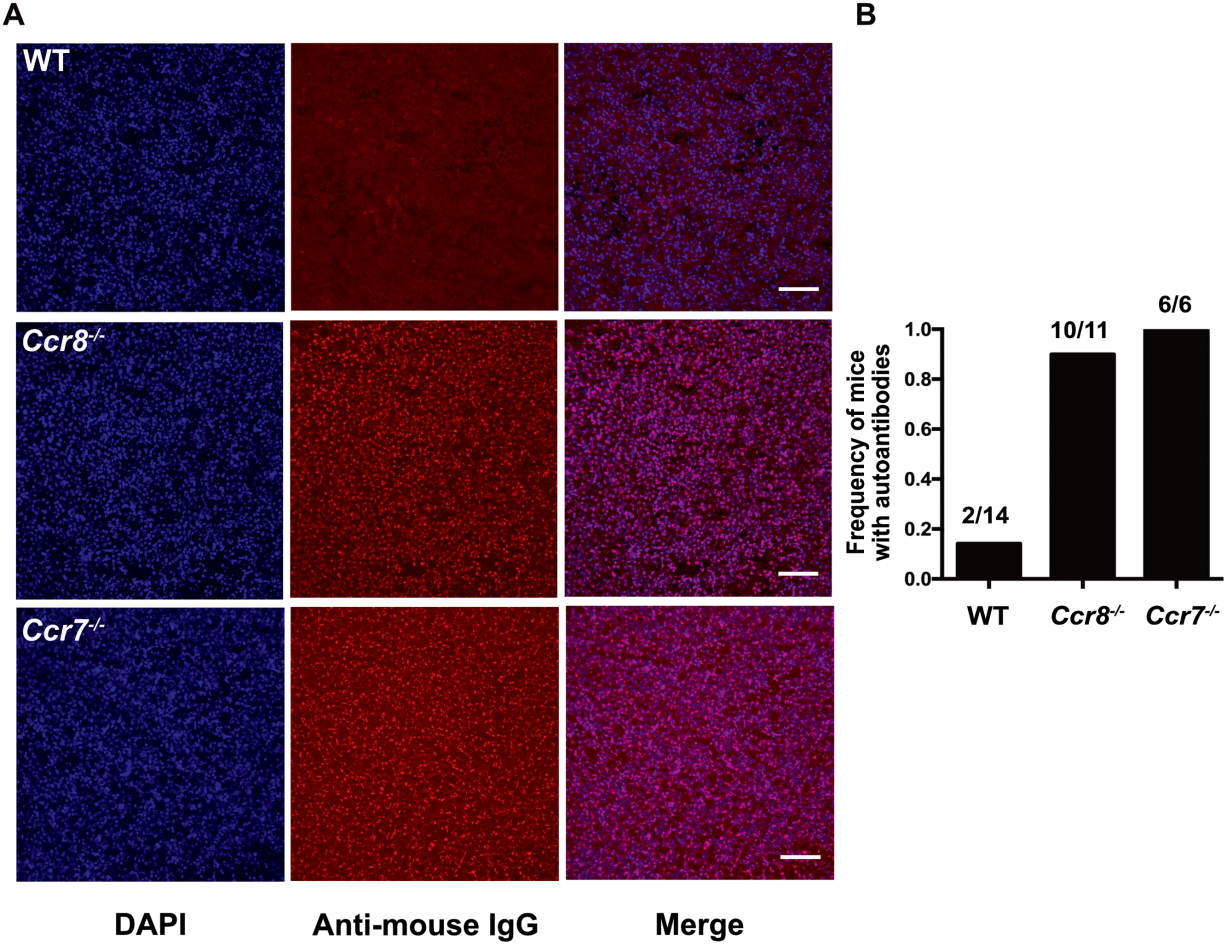
CCR8 deficiency results in increased production of autoantibodies. (A) Representative immunofluorescence images of kidney cryosections from *Rag2*^*-/-*^ mice immunostained with serum from 10–13 month old wild-type (WT) or *Ccr8*^*-/-*^ mice, or from 5–11 months old *Ccr7*^*-/-*^ mice. The presence or absence of autoantibodies was detected with anti-mouse IgG (red) and nuclei were stained with DAPI (blue). Scale bars 100μm. Images were taken at 20X magnification. (B) The proportion of aged mice containing serum autoantibodies was calculated from immunostaining as in (A). *** *p*<0.0001 (Fisher’s exact test).

One consequence of impaired thymic central tolerance or peripheral tolerance mechanisms is an increase in auto-antibodies. To determine whether CCR8 deficiency resulted in auto-reactive antibodies, we analyzed serum from *Ccr8*^*+/+*^ and *Ccr8*^*-/-*^ mice between 10–13 months of age. Anti-nuclear autoantibodies were present in *Ccr8*^*-/-*^ mice at a similar frequency to those detected in autoimmune *Ccr7*^*-/-*^ mice (Fig 6A and 6B). Thus, although our data do not support a role for CCR8 in thymocyte differentiation or negative selection, self-tolerance was broken in *Ccr8*^*-/-*^ mice, which likely reflects a role for CCR8 in peripheral tolerance mechanisms, as discussed below [30].

## Discussion

The establishment of central tolerance in the thymus is critical for preventing T-cell mediated autoimmune diseases. mTECs and DCs present highly diverse self-antigens to developing thymocytes in the medulla; strong TCR reactivity to these self-antigens causes thymocytes to undergo negative selection, eliminating autoreactive cells from the repertoire [1]. Alternatively, strongly self-reactive thymocytes can be diverted to the Treg lineage, which is also critical for suppressing peripheral autoimmunity [46]. Because the numerous self-antigens presented in the medulla are expressed at low levels by rare medullary APCs [9,10,11], it is critical that post-positive selection thymocytes efficiently enter the medulla and migrate rapidly therein to encounter the full array of self-antigens that promote central tolerance prior to egress to the periphery. We and others previously showed that chemokine receptors, including CCR7 [16–18,41,47], CCR4 [20,48], and EBI2 [21], which are expressed by post-positive selection thymocytes, and whose ligands are expressed in the medulla, promote efficient medullary accumulation of SP thymocytes, rapid motility of medullary thymocytes, and/or interactions with medullary APCs, thus promoting negative selection and self-tolerance. Because we found that CCR8 was expressed by post-positive selection thymocytes, while the ligands CCL8 and CCL1 were expressed by medullary stromal cells, we anticipated CCR8 would also promote thymocyte medullary entry and central tolerance.

CCR8 played only a minor role in medullary accumulation of SP thymocytes. Although CCR8 deficiency did not impact the localization of DP and SP thymocyte subsets as detected by immunofluorescence at steady-state, two-photon imaging of purified CD4SP cells migrating in thymic slices revealed a subtle defect in medullary enrichment. This is consistent with the observed trend of decreased medullary to cortical area observed in *Ccr8*^-/-^ thymi. Given that CCR8 is expressed by ~35% CD4SP cells, mainly in the SM and M1 maturation subsets, it remains possible that CCR8 has a more profound impact on medullary accumulation of these CD4SP subsets. Thus, we find that CCR8 contributes to the medullary entry of some post-positive selection CD4SP thymocytes, but its role is likely partially masked by expression on a fraction of CD4SP cells, and the more significant impact of CCR4 and CCR7 on overall CD4SP medullary entry [18,20].

Despite our hypothesis, we found no evidence to support a role for CCR8 in thymocyte differentiation or selection. CCR8 deficiency did not notably impact the ability of polyclonal or OT-II monoclonal thymocytes to undergo negative selection to endogenous self-antigens or the model OVA TRA, respectively. We previously observed that CCR4 and EBI2 were required for weak negative selection of OT-II thymocytes responding to endogenous ligands in C57BL/6J mice [20,21]. Notably, CCR8 was not required for this weak negative selection. Although CCR8 deficiency did not alter the cellularity or subset distribution of thymocytes at steady state or in the competitive context of mixed bone marrow chimeras, as would be expected if CCR8 were required for negative selection, CCR8 deficiency did result in increased cellularity of OT-II thymocytes in the context of bone marrow chimeras. However, CCR8 deficiency did not globally increase proliferation of OT-II thymocytes in these experiments, and we found no evidence that CCR8 generally impaired survival of thymocytes in a TCR-independent manner. Thus, it is unclear why increased thymocyte cellularity was observed in CCR8 OT-II chimeras. It is possible that CCR8 could impact survival, differentiation or migration of a hematopoietic progenitor that gives rise to thymocytes; however gene expression profiling does not indicate expression of CCR8 on any hematopoietic progenitors, including lymphoid progenitors [49], and if this were the case, increased cellularity of polyclonal *Ccr8*^-/-^ thymocytes would be expected. The increased thymocyte cellularity of *Ccr8*^-/-^ OT-II chimeras was not consistent with a negative selection defect, as the presence of the OVA TRA induced an equivalent reduction of cellularity to *Ccr8*^+/+^ OT-II chimeras. Thus, our data indicate that CCR8 is dispensable for the induction of central tolerance in the thymus.

Although central tolerance was intact, autoantibodies were present in the serum of aged *Ccr8*^*-/-*^ mice. A recent study demonstrated that CCR8 signaling via CCL1 engagement promotes the immunosuppressive activity of peripheral FOXP3^+^ Treg [30]. This study also showed that CCL1 was upregulated by FOXP3^+^ Treg in the CNS in an EAE mouse model of multiple sclerosis, driving a feed-forward loop in which CCR8 expression was upregulated, in turn promoting Treg-mediated suppression of autoimmunity. Consistent with this study, other groups have suggested that CCR8 promotes Treg-mediated immunosuppression in the periphery [28,29]. Therefore, because we find that negative selection and Treg induction are intact in *Ccr8*^*-/-*^ mice, the presence of serum autoantibodies in these mice likely reflects a requirement for CCR8 in maintaining peripheral tolerance mechanisms, likely through activation of FOXP3^+^ Treg. Altogether, our studies demonstrate that CCR8 is largely dispensable for thymocyte differentiation, negative selection, and thymic central tolerance.

## Supporting information

S1 Fig Gating scheme for FACS purification of thymocyte and thymic stromal cell subsets. (A) The gating scheme for FACS purification of DN, DP and SP thymocyte subsets is depicted. Cells were pre-gated for live, single cells. Lineage consisted of antibodies against B220, Gr-1, Mac-1, NK1.1 and Ter119, CD11c, and TCRγδ. (B) The gating scheme for FACS purification of thymic stromal cell subsets is depicted. Sromal cells were pre-gated for live cells. TEC (MHCII^+^ EpCAM^+^ CD11c^-^) were subdivided into cTEC (CD45^-^UEA-1^-^Ly51^+^), mTEC^hi^ (UEA-1^+^ MHCII^hi^ CD80^hi^), and mTEC^lo^ (UEA-1^+^ MHCII^lo^ CD80^lo^) subsets. Fibroblasts (CD45^-^ MHCII^-^ Ter119^-^ CD31^-^) and DCs (MHCII^+^ CD11c^+^ CD80^+^), which were subdivided into Sirpα^+^ and Sirpα^-^ subsets, were gated as indicated.
(PDF)

S2 Fig CCR8 expression by maturation subsets of CD4SP thymocytes in polyclonal *Ccr8*^*+/+*^ and monoclonal *Ccr8*^*+/+*^ OT-II mice. (A-B) Representative flow cytometric profiles showing sequential gating to identify CD4SP maturation subsets defined by CD69 and MHCI expression, along with cell surface CCR8 expression by each CD4SP subset in polyclonal *Ccr8*^*+/+*^ mice (A) and OT-II TCR transgenic mice (B).
(PDF)

S3 Fig CCR8 deficiency does not impact maturation, selection, proliferation or survival of OT-II thymocytes. (A) Cellularity of the indicated thymocyte subsets was determined for each bone marrow chimera group shown in Fig 4. (B) Two-way ANOVA was used to determine whether thymocyte subset cellularity was significantly impacted by CCR8 genotype, OVA expression, or the interaction of these two factors in the OT-II bone marrow chimeras. (C) The percentages of *Ccr8*^+/+^ and *Ccr8*^-/-^ OT-II thymocyte subsets were quantified based on flow cytometric analysis for the bone marrow chimera recipients shown in Fig 4. (D) Graphs display the percentage of *Ccr8*^+/+^ and *Ccr8*^-/-^ OT-II thymocyte subsets deleted in the presence of the OVA TRA in bone marrow chimera recipients shown in Fig 4. Percent deletion was calculated as the percent decrease in cellularity between OVA^-^ and RIP-mOVA^+^ recipients for the indicated subsets and genotypes from data as in A. (E) Representative flow cytometric plots showing DNA content, as assessed by intracellular staining with propidium iodide, used to determine the frequency of proliferating cells (gated for cells in S/G2/M). (F) Thymocytes from the OT-II chimeras in Fig 4 were analyzed to determine if CCR8 deficiency resulted in increased proliferation of thymocyte subsets. The percentages of the indicated thymocyte subsets in cell cycle (S/G2/M) were quantified by flow cytometry based on DNA content, as in (E). Graphs in A, C, D and F depict means + SEM compiled from the two independent experiments shown in Fig 4, with a total of n = 6 OT-II *Ccr8*^*+/+*^ → RIP mOVA^-^; n = 5 OT-II *Ccr8*^*+/+*^ → RIP mOVA^+^; n = 6 OT-II *Ccr8*^*-/-*^ → RIP mOVA^-^; n = 6 OT-II *Ccr8*^*-/-*^ → RIP mOVA^+^. (G) Quantification of the percent of *Ccr8*^*+/+*^ and *Ccr8*^*-/-*^ CD4SP thymocytes that were viable, as assessed by flow cytometric identification of PI^-^ AnnexinV^-^ cells, after incubation at 37°C, 5% CO_2_ for 24 hours in the presence or absence of CCL8. Graphs depict means + SEM from two independent experiments, with three technical repeats per experiment.
(PDF)

S4 Fig CCR8 deficiency does not impact the velocity or path straightness of CD4SP thymocytes. (A) Velocity and (B) straightnes of *Ccr8*^*+/+*^ and *Ccr8*^*-/-*^ CD4SP thymocytes migrating on live pCX-EGFP thymic slices were quantified from tracked cells. Data are compiled from CD4SP cells migrating in 13 slices, from a total of three biologically independent imaging experiments. Each dot represents the velocity (A) or path straightness (B) of a single tracked cell. Numbers indicate mean values, and the bar and whiskers indicate mean + SEM. NS: not significant (paired Student’s *t*-test). n = 100 *Ccr8*^*+/+*^ thymocytes; n = 94 *Ccr8*^*-/-+*^ thymocytes. See also S1 Movie.
(PDF)

S1 Movie CCR8 promotes medullary enrichment of CD4SP thymocytes. Two-photon time-lapse video microscopy of *Ccr8*^+/+^ (pink) and *Ccr8*^-/-^ (green) CD4SP thymocytes migrating in a pCX-EGFP live thymic slice (cyan). Frames were acquired at 15s intervals. A maximum intensity projection through 40μm is shown. These videos correlate with Fig 5A.
(MP4)
