# Supplementary figures and images for: CCR8 is expressed by post-positive selection CD4-lineage thymocytes but is dispensable for central tolerance induction
Source: PLoS One. 2018 Jul 19;13(7):e0200765. doi: 10.1371/journal.pone.0200765 (PMC6053179; doi:10.1371/journal.pone.0200765)

**A**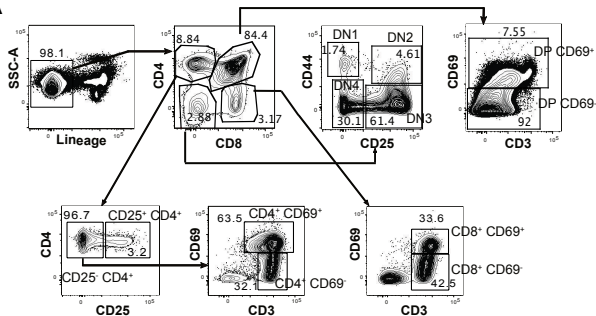**B**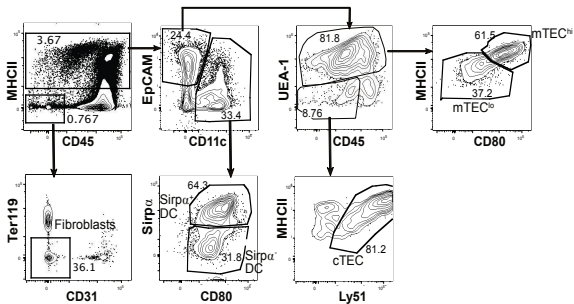

Supplement: S1 Fig — (A) The gating scheme for FACS purification of DN, DP and SP thymocyte subsets is depicted. Cells were pre-gated for live, single cells. Lineage consisted of antibodies against B220, Gr-1, Mac-1, NK1.1 and Ter119, CD11c, and TCRγδ. (B) The gating scheme for FACS purification of thymic stromal cell subsets is depicted. Sromal cells were pre-gated for live cells. TEC (MHCII+ EpCAM+ CD11c-) were subdivided into cTEC (CD45-UEA-1-Ly51+), mTEChi (UEA-1+ MHCIIhi CD80hi), and mTEClo (UEA-1+ MHCIIlo CD80lo) subsets. Fibroblasts (CD45- MHCII- Ter119- CD31-) and DCs (MHCII+ CD11c+ CD80+), which were subdivided into Sirpα+ and Sirpα- subsets, were gated as indicated. (PDF) [file pone.0200765.s001.pdf]

## A WT

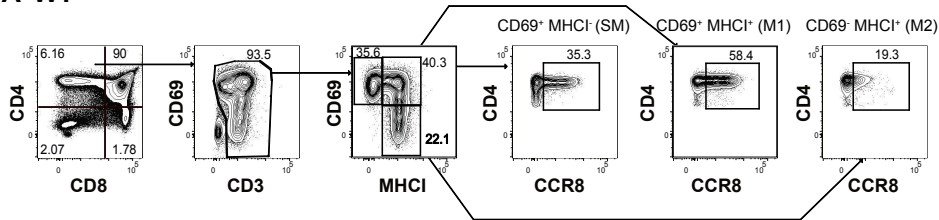

## B OT-II

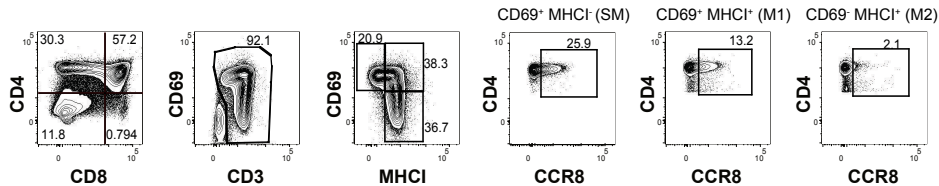

Supplement: S2 Fig — (A-B) Representative flow cytometric profiles showing sequential gating to identify CD4SP maturation subsets defined by CD69 and MHCI expression, along with cell surface CCR8 expression by each CD4SP subset in polyclonal Ccr8+/+ mice (A) and OT-II TCR transgenic mice (B). (PDF) [file pone.0200765.s002.pdf]

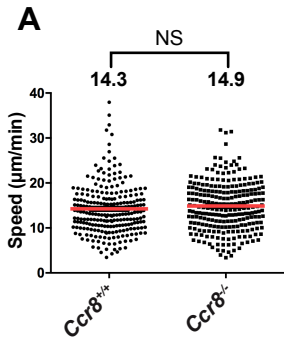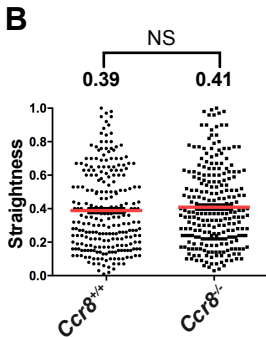

Supplement: S4 Fig — (A) Velocity and (B) straightnes of Ccr8+/+ and Ccr8-/- CD4SP thymocytes migrating on live pCX-EGFP thymic slices were quantified from tracked cells. Data are compiled from CD4SP cells migrating in 13 slices, from a total of three biologically independent imaging experiments. Each dot represents the velocity (A) or path straightness (B) of a single tracked cell. Numbers indicate mean values, and the bar and whiskers indicate mean + SEM. NS: not significant (paired Student’s t-test). n = 100 Ccr8+/+ thymocytes; n = 94 Ccr8-/-+ thymocytes. See also S1 Movie. (PDF) [file pone.0200765.s004.pdf]
