# Supplementary material for: CCR8 is expressed by post-positive selection CD4-lineage thymocytes but is dispensable for central tolerance induction
Source: PLoS One. 2018 Jul 19;13(7):e0200765. doi: 10.1371/journal.pone.0200765 (PMC6053179; doi:10.1371/journal.pone.0200765)

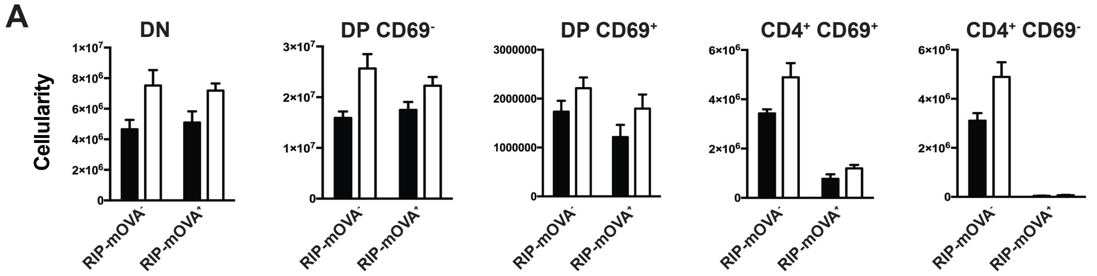

B

Significance determined by two-way ANOVA

Factors tested	DN	DP CD69-	DP CD69+	CD4+ CD69+	CD4+ CD69-
OVA+ vs OVA-	n.s.	n.s.	n.s.	*	*
WT vs *Ccr8*-/-	*	*	*	****	****
Interaction	n.s.	n.s.	n.s.	n.s.	n.s.

Ccr8+/+ OT-II

Ccr8-/- OT-II

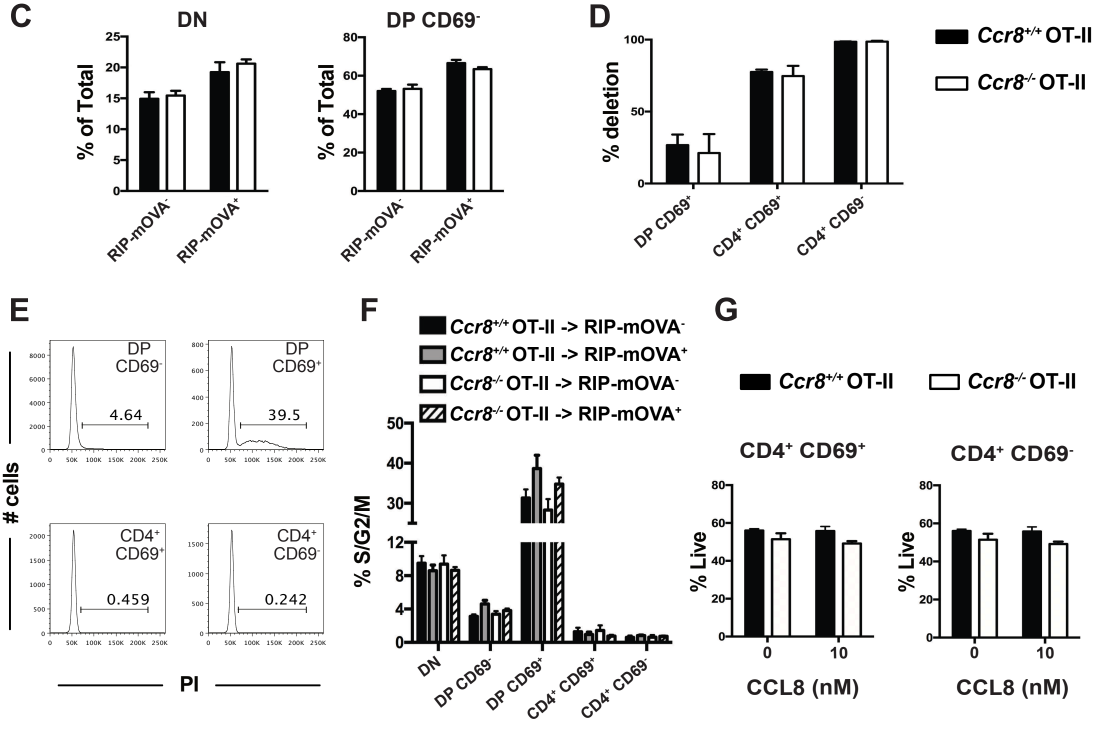

Supplement: S3 Fig — (A) Cellularity of the indicated thymocyte subsets was determined for each bone marrow chimera group shown in Fig 4. (B) Two-way ANOVA was used to determine whether thymocyte subset cellularity was significantly impacted by CCR8 genotype, OVA expression, or the interaction of these two factors in the OT-II bone marrow chimeras. (C) The percentages of Ccr8+/+ and Ccr8-/- OT-II thymocyte subsets were quantified based on flow cytometric analysis for the bone marrow chimera recipients shown in Fig 4. (D) Graphs display the percentage of Ccr8+/+ and Ccr8-/- OT-II thymocyte subsets deleted in the presence of the OVA TRA in bone marrow chimera recipients shown in Fig 4. Percent deletion was calculated as the percent decrease in cellularity between OVA- and RIP-mOVA+ recipients for the indicated subsets and genotypes from data as in A. (E) Representative flow cytometric plots showing DNA content, as assessed by intracellular staining with propidium iodide, used to determine the frequency of proliferating cells (gated for cells in S/G2/M). (F) Thymocytes from the OT-II chimeras in Fig 4 were analyzed to determine if CCR8 deficiency resulted in increased proliferation of thymocyte subsets. The percentages of the indicated thymocyte subsets in cell cycle (S/G2/M) were quantified by flow cytometry based on DNA content, as in (E). Graphs in A, C, D and F depict means + SEM compiled from the two independent experiments shown in Fig 4, with a total of n = 6 OT-II Ccr8+/+ → RIP mOVA-; n = 5 OT-II Ccr8+/+ → RIP mOVA+; n = 6 OT-II Ccr8-/- → RIP mOVA-; n = 6 OT-II Ccr8-/- → RIP mOVA+. (G) Quantification of the percent of Ccr8+/+ and Ccr8-/- CD4SP thymocytes that were viable, as assessed by flow cytometric identification of PI- AnnexinV- cells, after incubation at 37°C, 5% CO2 for 24 hours in the presence or absence of CCL8. Graphs depict means + SEM from two independent experiments, with three technical repeats per experiment. (PDF) [file pone.0200765.s003.pdf]
